# The impact and treatment of obesity in kidney transplant candidates and recipients

**DOI:** 10.1186/s40697-015-0059-4

**Published:** 2015-08-01

**Authors:** Gabriel Chan, Pierre Garneau, Roy Hajjar

**Affiliations:** Département de Chirurgie, Université de Montréal and Service de Transplantation Rénale, Hôpital Maisonneuve-Rosemont, Montreal, Canada; Département de Chirurgie, Université de Montréal, Hôpital Sacre-Cœur de Montréal, Montreal, Canada

**Keywords:** Obesity, Chronic kidney failure, Bariatric surgery, Pharmacokinetics, Kidney transplantation

## Abstract

The prevalence of obesity in patients with chronic kidney failure and renal transplant candidates has paralleled the epidemic in the general population. The associated risks of surgical complications and long-term cardiovascular death are significant: most transplant centers consider obesity a relative contra-indication for transplant.

Few studies have focused on conservative weight loss strategies in transplant patients. Studies using administrative databases have found that only a minority of wait-listed patients lose weight and with no apparent benefit to transplant outcomes. The only clinical trial in this area found that an intensive weight-loss program had significantly better success (to listing) than self-directed weight loss. However, only a minority that succeeded with the help of a program (36 %), while the “diet and exercise” group had negligible results.

Laparoscopy has radically shortened the recovery time and decreased the complications associated with bariatric surgery. Reports in transplant patients, who were previously deemed too medically complex, have demonstrated a dramatic and rapid weight loss. The only randomized clinical trial in patients with CKD, which compared sleeve gastrectomy to best medical care clearly favoured the surgical arm for weight loss, but was too small to assess other outcomes. The emerging experience is small but quite promising.

Surgical complications and the effect on immunosuppression remain the chief concerns regarding the use of bariatric surgery in transplant patients. Rigorous prospective studies will be essential to properly evaluate the expected weight loss and the effect on pharmacokinetics of immunosuppressive medications. A routine role for bariatric surgery in transplantation would require evidence of improvements in patient-important outcomes and evidence of safety.

## Abrégé

La prévalence de l’obésité chez les patients atteints d’insuffisance rénale chronique et les candidats à la greffe rénale a atteint les mêmes proportions épidémiques que dans l’ensemble de la population. Les risques associés, tels que les complications chirurgicales et la mortalité cardiovasculaire à long terme, sont considérables: la plupart des centres de greffe considèrent l’obésité comme une contre-indication relative à la greffe.

Peu d’études portent sur les stratégies visant une perte de poids minimale chez les greffés. Des études fondées sur des bases de données administratives ont révélé que seule une minorité de patients inscrits sur les listes d’attente perd du poids, et qu’aucune amélioration apparente n’a été notée dans les résultats de la greffe. La seule étude clinique dans ce domaine démontre qu’un programme intensif de perte de poids contribuait davantage à une réussite que la perte de poids autogérée. Toutefois, seule une minorité a réussi avec l’aide d’un programme (36 %), alors que le groupe qui se concentrait sur un régime alimentaire et sur l’exercice a obtenu des résultats négligeables.

La laparoscopie a réduit radicalement le temps de rétablissement et a entraîné la diminution des complications associées à la chirurgie de l’obésité. On rapporte une perte de poids importante et rapide chez des patients dont le cas avait précédemment été jugé trop complexe du point de vue médical. Le seul essai clinique aléatoire effectué sur des patients atteints d’IRC, lors duquel on comparait la gastrectomie longitudinale aux soins médicaux d’excellence, favorisait manifestement le recours à la chirurgie dans la perte de poids. Elle était toutefois trop restreinte pour évaluer d’autres résultats. L’expérience est certes limitée, mais plutôt prometteuse.

Les complications chirurgicales et l’effet sur l’immunosuppression demeurent les préoccupations principales concernant la chirurgie de l’obésité chez les greffés. Des études prospectives rigoureuses seront essentielles afin d’évaluer adéquatement la perte de poids espérée, de même que les effets des médicaments immunosuppresseurs sur la pharmacocinétique. Il faudrait que soient notées des preuves d’une amélioration des résultats concernant le patient, de même que des preuves de sûreté, pour que la chirurgie de l’obésité lors des greffes devienne une procédure de routine.

## What was known before

Obesity is prevalent in patients with CKD, including transplant candidates, is a barrier to transplantation, and is associated with worse outcomes after transplantation.

## What this adds

We reviewed the literature and summarized studies on the impact of obesity on listing for transplantation and outcomes after transplantation. We review bariatric surgical techniques and summarize literature on bariatric surgery in transplant candidates and recipients.

## Background

Obesity has become prevalent and presents a difficult dilemma for kidney transplant programs [1]. The major obesity-associated risk is a higher rate of cardiovascular death, thus most transplant centers restrict access to the transplant waiting list according to a body mass index (BMI) limit, usually in the order of 35 to 40 kg/m^2^ [2]. This type of restrictive policy means that many patients are ineligible for transplantation if they cannot lose the excess weight. Of course it is no secret that voluntary weight loss is already a desperate challenge in the general population. To attempt this while on hemodialysis would seem even more daunting.

Until now, scant resources have been dedicated to the treatment of obesity by kidney transplant programs. Correspondingly, there is a paucity of transplant research into weight management strategies. Some transplant specialists have commented that managing a comprehensive weight loss programme is beyond the resources and scope of most transplant centers [3].

In the general population, bariatric surgery has demonstrated dramatic weight loss results in addition to significant improvements to the diabetic profile [4]. In the era of the laparoscopic approach, the outcomes of bariatric surgery have greatly improved with fewer complications and a much shorter length-of-stay. Whether similar benefits with an acceptable risk profile can be demonstrated in the transplant population, candidate or recipient, remains to be demonstrated. This review presents the current published experience of bariatric surgery in the kidney transplant population. The Pubmed database was searched using the following criteria: kidney transplantation, kidney disease, candidate, recipient, obesity, weight loss, bariatric, sleeve gastrectomy, gastric band, gastric bypass and duodenal switch. The reference lists of selected publications were also cross referenced to complete the literature search.

### Risks associated with obesity in kidney transplant patients

The kidney transplant operation is without a doubt technically more difficult in the obese recipient, in particular limiting the operative exposure of the external iliac vessels and the bladder in the properitoneal and retroperitoneal spaces. This is reflected in the relatively longer operative times required in obese recipients [5, 6], a higher rate of surgical complications [7, 8], increased surgical site infections [5, 6, 9, 10] and more lymphoceles [6]. The length of stay, which is a surrogate measure of a complicated recovery, is significantly longer [5, 9, 11]. The overall impact of post-operative complications such as surgical site infection should not be under-estimated, as it has been shown to be significantly associated with graft loss [12].

Worse graft-related outcomes have been associated with obesity. Several studies have found an increased risk of delayed graft function in patients with a BMI >35 kg/m^2^ [11, 13], and other studies have shown progressive increased risk with increased BMI [14, 15]. Furthermore, risk of acute rejection has been found to be increased in obese recipients [8, 11, 16]. Several hypotheses could explain the higher rate of rejection, including under-dosing of immunosuppression, altered pharmacokinetics or increased risk of infections that require more frequent adjustment of immunosuppression levels.

Obese recipients experience exacerbations of all aspects of the metabolic syndrome: hypertension, diabetes and dyslipidemia [13, 17]. The risks of congestive heart failure, atrial fibrillation and myocardial infarction increase with each BMI quartile [18], and worse proteinuria has been also found in obese recipients [17].

Not surprisingly the culmination of all these risks is a significantly diminished survival of both the graft and the patient. Several large database studies have found that obese recipients have a significantly lower graft survival [15, 19, 20]. The relative risk of graft loss at 5 years was 1.385 [confidence interval (CI) = 1.300 - 1.551] as compared to the non-obese population in the United States Renal Data System (USRDS) database [13]. A retrospective review of the national database in the Netherlands found that overall patient survival was significantly worse at 5 years in obese recipients (81 %) versus non-obese recipients (89 %) [19]. An analysis of the USRDS database from 1988 – 1997 found that a BMI > 36 kg/m^2^ was associated with poor survival outcomes on both sides of the coin: a higher mortality with a functioning graft, and death-censored graft loss [13]. In this study, patient mortality was primarily attributed to cardiovascular and infectious diseases. In another study of the USRDS (1995–1999), it was found that the survival benefit of transplantation versus remaining on dialysis was lost above a BMI of 41 kg/m^2^ [21]. A more recent study of the same USRDS database (1995 – 2007) was able to demonstrate a similar survival benefit at one year for the morbidly obese cohort in the living-donor transplantation setting (versus non-morbidly obese recipients) [22]. For standard criteria donor transplantation, this last study found a diminished survival benefit (48 % relative reduction of risk versus remaining on dialysis versus > 66 % in non-obese) in morbidly obese recipients. One weakness of this study is that one year may be too short a period of follow-up for the impact of cardiovascular disease to manifest itself. It should be noted that not all studies have shown a statistically significant risk of mortality [15, 16, 23]. A comprehensive review of the risks and complications associated with obesity in transplantation was published recently [24]. A dangerous tendency for worse outcomes in obese patients has been identified and warrants caution and further investigation.

### Weight gain post-transplantation

If the treatment of obesity is deferred until after kidney transplantation, it may actually become more difficult to manage. Early weight gain is common amongst recipients with a doubling of the number of obese patients from 5.6 % pre-transplantation to 11.4 % one year after transplantation. [20]. Post-transplant weight gain may be even more dangerous than pre-transplant obesity, as this study showed that it was associated with a higher risk of death and graft failure (relative risk (RR) = 1.39 [CI: 1.05 - 1.86] and 1.39 [CI: 1.10 – 1.74]), versus with pre-transplant BMI (RR of 1.22 [CI: 0.86 – 1.74] and 1.34 [1.02 – 1.77], respectively). Not surprisingly, post-transplant weight gain has also been associated with increased post-operative complications [25], worsening of the metabolic syndrome and a reduction in long-term graft function [26, 27], increased graft loss [28] and worse patient and graft survival [27]. Post-transplant weight gain appears to be independent of steroids or graft function [29, 30]. Whether post-transplant weight gain or any of the associated risks can be reversed with an effective weight management strategy remains to be investigated. Logically, initiating a weight management strategy prior to transplantation could lead to decreased complications of the transplant operation and diminish the risk of post-transplantation weight gain. Finally, it should be relevant to consider a possible change in motivation or compliance with weight loss interventions once a kidney transplant has been realized, whether positive or negative.

### Conservative weight loss in transplant candidates

The current literature regarding weight loss prior to transplantation is limited. In the post-transplantation setting, there are no published studies. Database studies have shown that fewer than 10 % of potential candidates lose some weight when requested for listing and even fewer (5 %) attain the target BMI of < 30 [31]. Another study found that 30 % of obese candidates on the waiting list had a decrease in the BMI to below 35 kg/m^2^ at transplantation [32]. Unfortunately, the patients who had lost weight to gain access to the waiting list, had the highest risk of rapidly re-gaining weight in the early period post-transplantation, without any significant benefit to survival or graft outcomes [32]. The main weakness of these database studies is that there is usually no way to ascertain the nature of the weight loss, whether intentional or due to another acute co-morbid disease. There is also no indication of whether anthropometric measures were routinely updated in the database. There is only one published prospective study that describes a clinical trial of a comprehensive weight loss program for transplant candidates [33]. The weight loss program, including regular exercise, nutrition, behavioural therapy and Orlistat, an oral lipase inhibitor, was compared to self-directed diet and exercise in the non-consenting subjects. The program required monthly surveillance for six months then bi-annually thereafter. The results found that at two years significantly more patients had successful weight loss with acceptance to the waiting list in the weight loss program group (35 %) versus the self-directed ‘diet and exercise’ control group (6 %). The mean weight of the intervention group dropped to 94.6 kg versus 101.0 kg in the control group at two years follow-up. It should be noted that the actual weight loss achieved was less than 10 % over a two year study period. There is no doubt that significant voluntary weight loss in chronic renal failure (CRF) patients, even with intensive support, is difficult to achieve. The long-term sustainability, widespread applicability and actual benefits of a comprehensive, labour-intensive weight loss program in the transplant population are still unknown.

### Bariatric surgery and transplantation

The National Institutes of Health indications for bariatric surgery are a BMI ≥40 mg/kg^2^ alone or, a BMI > 35 mg/kg^2^ with at least one co-morbid disease, including hypertension, diabetes, sleep apnea, arthropathy, coronary artery disease or dyslipidemia [34]. In addition, patients are required to have made at least one attempt at a structured program of weight loss. Given that the two most common causes of renal failure are diabetes and hypertension, the vast majority of obese patients with chronic kidney disease fit the criteria for bariatric surgery at a BMI of 35 kg/m^2^.

**Fig. 1:**
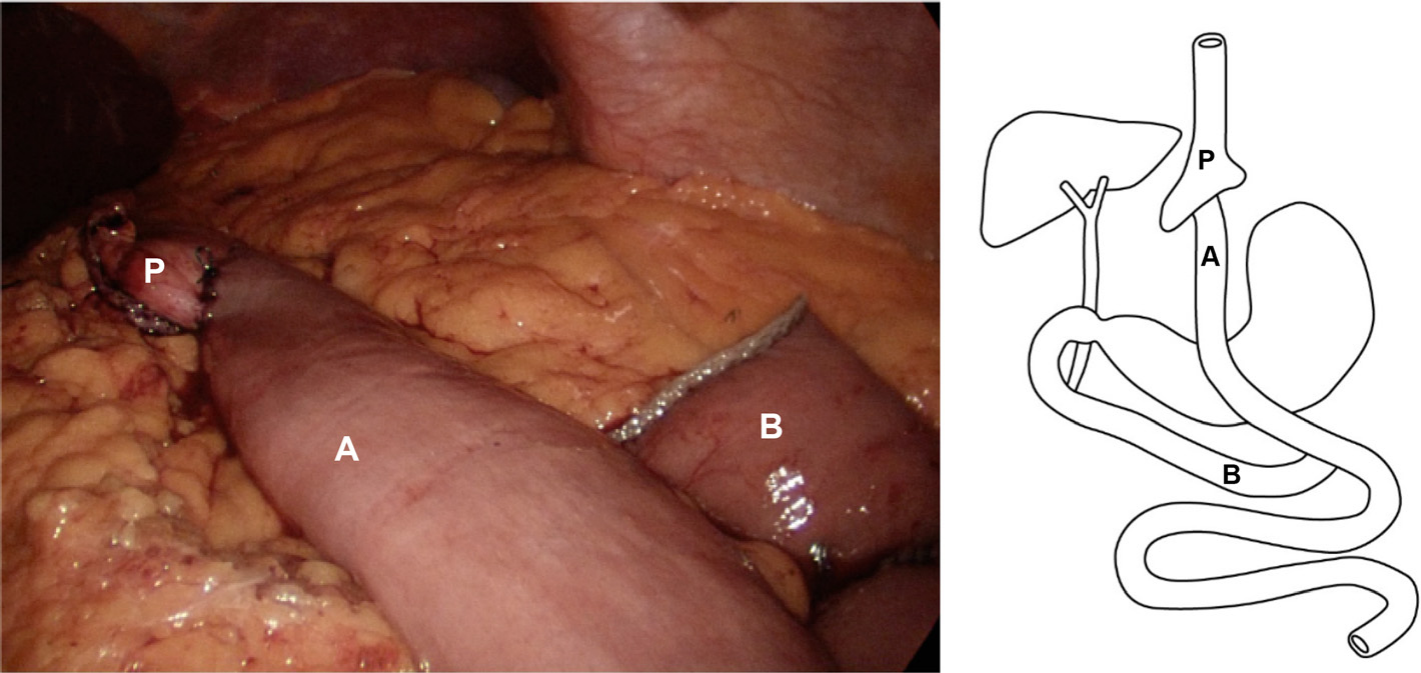
Gastric Bypass. The alimentary limb (A) has been anastomosed to the gastric pouch (P). The biliopancreatic limb (B) will be reconnected distally onto the alimentary limb.

**Fig. 2:**
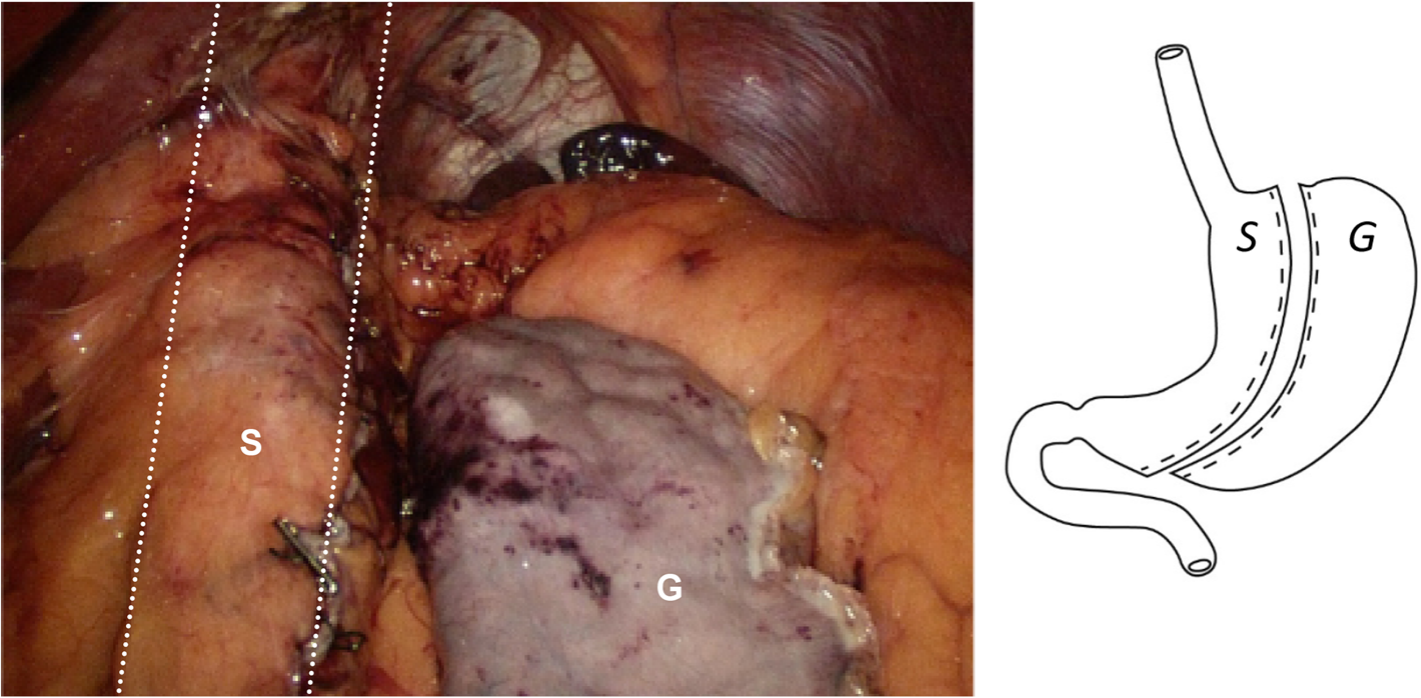
Sleeve Gastrectomy. The sleeve (*S)* is outlined by the dashed lines*.* The resected gastrectomy specimen (*G*) is seen on the right.

Presently, the most common laparoscopic bariatric techniques are the Roux-en-Y gastric bypass (LGB) (Fig. 1), the duodenal switch (or bilio-pancreatic diversion) (LDS) and the sleeve (or vertical) gastrectomy (LSG) (Fig. 2). The gastric band procedure has fallen out of favour due to multiple factors including the need for manipulations post-implantation, the risk of technical complications and the emergence of the LSG. The characteristics of the four types of bariatric surgeries are summarized in Table 1. The minimally invasive or laparoscopic technique has transformed bariatric surgery by dramatically decreasing operative times, post-operative complications and length of stay, with similar weight loss outcomes. The LGB and the LDS both use restrictive and malabsorptive mechanisms. The restrictive mechanism reduces the capacity of the stomach to as little as 30 ml. and induces early satiety. The malabsorptive mechanism works by bypassing a significant portion of the small intestine and decreasing the number of calories absorbed into the body. The LSG is a purely restrictive procedure that involves the resection of the majority of the stomach to leave a remnant gastric tube along the lesser curve between the esophagus and the duodenum. The technique was traditionally the first step of the LDS, which was occasionally performed in two separate surgical stages for medically high risk patients. This experience found that in many cases LSG alone was sufficient to induce a significant weight loss. The use of LSG has expanded widely over the last decade [35] likely due to its simplicity and has made the gastric band procedures obsolete. The hormonal mechanism of weight loss has been associated with the bypass or resection of the stomach, and the subsequent loss of the influence of ghrelin, the “hunger” hormone. After bariatric surgery, patients will report a loss of appetite that was previously excessive or insatiable.

**Table 1:** Summary of the characteristics and the associated risks of different bariatric operations

Operation	Weight loss mechanism			Volume of gastric pouch (ml)	Length of bypassed small bowel (cm)	Expected EWL (%)	Physiological risks in the general population	Post-bariatric surgery nutritional supplementation
	Restrictive	Malabsorption	Hormonal					
Sleeve gastrectomy	+		+	100 - 150	0	50 - 60	GERD, B12-deficiency anemia	MV, Ca
Gastric bypass	+	+	+	15 - 30	130 - 180	60 - 70	Peptic ulcers, anemia, osteoporosis, dumping syndrome, kidney stones	MV, B12, Fe, Ca, vit D
Bilio-pancreatic diversion and duodenal switch	+	+	+	100 - 250	250 - 300	70 - 80	Diarrhea/steatorrhea, anemia, vitamin deficiencies	MV, B12, Fe, Ca, vitamins A, D, E
Adjustable gastric band	+			30	0	20 - 40	Band erosion or migration	Nil

The published literature of bariatric surgery in kidney transplant patients are almost all retrospective, comprising of database studies and institutional case series. The largest published series are from the open era of gastric bypass and are of historical interest. Modanlou *et al.* used the registry data of the USRDS, Organ Procurement and Transplantation Network and Medicare billing claims to identify patients who underwent bariatric surgery who had CRF or a kidney transplantation. One hundred and eighty-eight cases over 15 years were identified. The 30-day mortality after bariatric surgery in listed and transplanted patients was 3.5 %, due primarily to cardiac and infectious complications. The weight loss achieved was available in only 44 % of the cohort. The excess body weight loss was within the range of 31 – 61 %. The specific time period over which this weight loss was achieved was not included [36]. Alexander *et al.* published the institutional experience with a cohort of 41 patients in CRF, dialysis and renal transplant recipients undergoing open gastric bypass [37]. The average excess BMI lost by one year was 68 % with the majority of weight loss achieved by 12 months. In the historical context, open bariatric surgery resulted in significant weight loss, with a small but real risk of mortality. The adoption of the laparoscopic technique has improved radically upon the benefit/risk ratio.

Several case reports have described the weight loss achieved with banded gastroplasty procedures in transplant candidates and recipients [38–41]. However, gastric banding has fallen out of favour in the bariatric community [35] and is associated with supplemental risk of foreign body complications like erosion and migration [35, 42]. This type of procedure should only be considered for transplant patients with extreme caution.

### Laparoscopic bariatric surgery in the transplant candidate

More recently, several publications have reported the preliminary experiences with laparoscopic bariatric surgery (Table 2). Seven retrospective case series have included patients in the pre-transplant setting. The largest series reported 52 candidates who underwent LSG. The mean change in BMI achieved through surgical weight loss was −6.6 kg/m^2^ with the mean percentage of excess body weight loss (EBWL) of 29.8 % over a mean follow-up of 220 days. A slight majority of patients (55.8 %) in this cohort were able to achieve a BMI < 35 kg/m^2^ and activation on the waiting list [43]. Lin *et al.* included six CRF patients amongst a cohort of 26 patients with end-organ failure who were treated with LSG. In their entire cohort, including patients with renal or liver failure, the drop in BMI was 10.7 kg/m^2^ and 14.2 kg/m^2^, at 6 months and 12 months respectively [44]. The results specific to the renal failure patients were not specified. Takata *et al.* reported another series of end-organ failure patients, including 7 with end-stage renal disease (ESRD) who underwent LGB. All patients in this series were able to reach the BMI limit for transplantation at the University of California at San Francisco of 40 kg/m^2^ within a follow-up period of 3–18 months [45]. Unfortunately, these two series had heterogeneous populations mixing liver, lung and kidney patients, making generalizations to the CRF population difficult. MacLaughlin *et al.* published a series of nine CRF patients undergoing LSG and reported a BMI decrease of 8.4 kg/m^2^ at 6 months, representing a 43 % excess body weight loss [46]. Tariq *et al.* reported an experience of LGB in 7 ESRD patients. All of the patients were able to achieve sufficient weight loss for a BMI < 35 kg/m^2^ within 6 months of the bariatric surgery [47]. Another report of three gastric bypass patients, of which two were performed by laparoscopy, had a mean change in BMI of −8.7 kg/m^2^ at 3 months [48]. Finally, one case report has been published of robotic assisted gastric bypass in a hemodialysis patient which resulted in a drop in BMI of −12.5 kg/m^2^ at 3 months and −14.9 kg/m^2^ at ten months [49]. Clearly, the preliminary experiences with laparoscopic bariatric surgery in the kidney transplant candidate has shown rapid and vastly superior weight loss as compared to any previous published experiences of “medical weight loss”. The major limitations of these studies are the lack of conformity in reporting weight loss results. The weight loss was most often reported as a change in BMI, though some only reported the number who achieved the BMI limit for transplantation. Only one study reported the results as the EBWL. Furthermore, the study follow-up times have varied between 3 to 18 months. This heterogeneity coupled with the small study sizes unfortunately limits the general conclusions that can be drawn from these studies.

**Table 2:** Published clinical studies of laparoscopic bariatric surgery in kidney transplantation

Author (year)	Type of operation	n	CKD stage	Weight loss achieved
Freeman (2015)	LSG	52	V (47); IV (5)	Mean ΔBMI = −6.7 kg/m^2^, mean %EBWL = 29.8 %; %BMI < 35 = 55.8 %
Tariq (2013)	LGB	7	V	↓BMI < 35 at 6 months in 100 % cohort
Lin (2013)	LSG	6	V (5); IV (1)	Mean EBWL = 50 % at 12 months*
Proczko (2013)	LGB	3	V	Mean ΔBMI = −8.7 kg/m^2^ at 3 months
MacLaughlin (2012)	LSG	9	V (5)	Median EBWL = 43 %; median ΔBMI = −8.4 kg/m^2^ at 6 months
Takata (2008)	LGB	7	V	Mean EBWL 61 % at 9 months
MacLauglin RCT (2014)	LSG vs. BMC	5 vs. 6	III/IV	Mean ΔBMI: −12.0 vs −1.2 kg/m^2^ at 12 months
Golomb (2014)	LSG	10	Post-transplant	Mean EBWL = 75 % at 12 months
Szomstein (2010)	LGB/LSG	4/1	Post-transplant	EBWL > 50 % at 2 years in 100 % of cohort
Arias (2010)	LGB	5	Post-transplant	Mean ΔBMI = −11 kg/m^2^

*mean includes chronic liver failure patients

(CKD chronic kidney disease; LGB laparoscopic gastric bypass; LSG laparoscopic sleeve gastrectomy; BMI body mass index; EBWL excess body weight loss; RCT randomized clinical trail; BMC best medical care)

### Bariatric surgery in the transplant recipient

Three reports have been published describing the experience in the post-transplant setting (Table 1). Szomstein *et al.* reported a series of five recipients, including four who underwent LGB and LSG in the other. All patients in this series achieved greater than 50 % EBWL at two years with the change in BMI ranging between −13.6 kg/m^2^ to −37 kg/m^2^ [50]. Arias *et al*. reported 5 recipients with laparoscopic gastric bypass with a mean weight loss of −33.4 kg. Interestingly, these patients had gained on average 16.75 kg since their kidney transplantation [51]. Golomb *et al.* has published the most recent series of ten cases of LSG that occurred on average 6 years after transplantation. The median decrease in BMI was −13 kg/m^2^ with an average % EBWL of 75 % at one year. One patient failed weight control with sleeve gastrectomy and required conversion to bilio-pancreatic diversion at 14 months [52]. Similar to the experience in transplant candidates, the observed weight loss is very promising but limited by the small sample sizes.

The question regarding the ideal sequence between the bariatric surgery and the kidney transplantation, is probably more practical than theoretical. In an ideal situation, any treatment of obesity would occur at the earliest time, prior to transplantation. This would conceivably decrease the risk of operative complications and improve early graft function, in addition to treating diabetes and hypertension, increasing graft survival and lowering the risk of cardiovascular death in the long term. The average waiting time for a kidney graft in Canada is between 2 and 5 years depending on the province (2014 Canadian Organ Replacement Register Report - Treatment of End-Stage Organ Failure in Canada 2003–2012, available at www.cihi.ca), which should be sufficient time for a consultation with bariatric surgery, the operation, recovery afterwards and the subsequent weight loss. Bariatric surgery could possibly also negate post-transplant weight gain commonly observed [27, 28, 53]. Delaying bariatric surgery until after transplantation is the clinical alternative. Some specialists might argue that a crucial factor in long-term outcomes is the time spent on dialysis, thus even for an obese patient, the earlier a transplant can be performed, the better. However, delaying bariatric surgery would not address the technical complications associated with obesity at a kidney transplantation. Furthermore, a gastric leak, though an uncommon complication, could be more complicated to manage in a patient on immunosuppressive medication than in CRF. If sepsis were to develop it would be conceivable that lowering the immunosuppression would be necessary thereby increasing the risk for graft rejection. In considering the balance of benefit and risk, the transplant specialist needs to compare the sum of the operative risks of bariatric surgery in a CRF patient and the potentially improved risks of a kidney transplantation in a less-obese recipient *versus* the combined risks of a kidney transplantation in an obese candidate and the risks of bariatric surgery in an immunosuppressed patient.

### Prospective clinical trial of bariatric surgery in transplant candidates

Only one small prospective randomized trial has been published comparing laparoscopic sleeve gastrectomy and best medical care in stage 3/4 CKF patients. In the bariatric surgery arm, there was a vastly superior weight loss, with an adjusted between-group difference of −10.8 kg/m^2^ for the change in BMI. There were also important and statistically significant improvements in Anxiety/Depression scores, and in Physical Domain and Mental Domain scores of the SF-36 quality of life questionnaire. However, there was no difference between the two groups in measured renal function or proteinuria. Clearly, in addition to the dramatic weight loss, there are secondary benefits to quality of life for the patient. The authors did note that the recruitment was difficult and ultimately, the small sample size (n = 11) severely limited the strength of the study’s conclusions. Unfortunately, the hope that surgical weight loss could improve or stabilize renal failure was not realized in this small study [54].

### Secondary benefits of bariatric surgery

Diabetic parameters such as insulin requirements and Hb_A1C_ levels have been shown to substantially improve after bariatric surgery [4]. Amongst the published reports in kidney transplantation, the decrease in daily insulin requirements have varied from −24 % [46] to −88 % [55], with some patients no longer requiring any insulin at all [44, 45, 51, 55]. In the small randomized clinical trial, the observed inter-group difference was −36 units in the daily mean insulin dose, clearly favouring the bariatric arm [54]. The potential secondary benefit of eliminating the need of insulin may be just as dramatic and promising as the weight loss results for the transplant patient.

There could be a similar benefit to hypertensive control after bariatric surgery [45]. In one case series, all five patients, who previously required multiple medications, were able to reduce this to one or none after the surgery [51]. In another series, three out of seven patients were able to reduce the number of medications [45].

Perhaps the most intriguing of all secondary benefits, is the potential of improvement in residual renal function after bariatric surgery. One case report described a patient who underwent gastric bypass surgery and was able to discontinue dialysis three months post-operatively [56]. In the retrospective series of LSG of Lin et al., one patient had sufficient improvement of residual renal function that he was removed from the waiting list for transplantation [44]. Another study described a cohort of nine patients who had stabilized or improved their renal function post-bariatric surgery, including one complete pathological resolution of membranous glomerulonephritis, and two other patients with focal segmental glomerulosclerosis were able to stop dialysis [57]. A retrospective series of 25 patients with stage III CRF who underwent (an unspecified) bariatric surgery demonstrated an improvement in mean creatinine clearance by MDRD from 47.9 ml/min/1.73 m to 61.6 ml/min/1.73 m^2^ at 1 year [58]. However, the only prospective randomized study, as mentioned above, did not find any improvement to residual renal function as measured by body surface area-adjusted creatinine clearance, either by the MDRD or CKD-EPI formulae [46]. Larger prospective studies are required to identify the subset of patients who could potentially benefit with an improvement in the chronic renal dysfunction after bariatric surgery. The potential mechanisms of the weight loss related improvement of CRF could be the consequence of improvements to hypertensive or diabetic parameters, resolution of the chronic inflammatory state of obesity, or through the resolution of another obesity-related pathophysiology.

### Surgical complications of bariatric surgery in the transplant population

The safety of bariatric surgery in CRF patients or transplant recipients is one of the main concerns. A survey of the results reported in the published case series’ can at least provides a preliminary indication of its safety profile in this setting. Volume management appears to be a crucial aspect of the post-operative care and figures prominently in these reports. Acute kidney injury from a decrease in effective volume, especially in a pre-dialysis patient or transplant recipient [44, 46, 52] is a serious complication, and has to recognized and avoided. The margin between dehydration and fluid overload is much narrower than normally seen in the general population for which standard post-operative protocols used in bariatric surgery were intended. The education of the patient to avoid vomiting and to focus on hydration in the early period post-bariatric surgery should be emphasized. Furthermore, as the patient loses weight rapidly, hemodialysis parameters evolve rapidly and must be adjusted accordingly. Hypotension during dialysis can occur as these parameters can be difficult to adapt to [55]. Other reported complications are common or particular to the CRF patient, such as myocardial infarction [46] and compromised vascular access [46] Some complications are related to bariatric surgery such as gastric stricture [52], delayed gastric emptying [46] and gastric leak [46]. In all, four series’ did not report any post-operative complications [45, 47, 50, 51]. These preliminary studies point to areas that will require focus in future studies, including pre-operative cardiovascular assessment and close post-operative volume management. Of note, no mortalities have been reported, but obviously as with all the possible complications, is subject to a publication bias and limited the small study populations.

### Physiologic complications of bariatric surgery

The physiological changes related to bariatric surgery can lead to other complications and are another source of concern [34, 59, 60]. Malabsorptive operations, such as Roux-en-Y gastric bypass and the duodenal switch, can lead to hyperoxaluria, oxalate lithiasis and renal oxalosis which can in turn cause renal damage [60, 61]. Intestinal bypass operations are also complicated by the malabsorption of minerals and vitamins, including zinc, calcium, vitamin D, iron and B complex vitamins [62]. Other concerns have been expressed about the aggravation of calcium deficiency and hyperparathyroidism in bariatric patients undergoing renal replacement therapy [63]. None of these metabolic complications have been typically associated with sleeve gastrectomy, which uses only a restrictive mechanism. Finally, a major concern particular to transplant specialists is the potential effect of bariatric surgery on the pharmacokinetics of immunosuppressive medications.

### General pharmacokinetics post-bariatric surgery

The effect of bariatric surgery on the pharmacokinetics of common medications consist of alterations to bioavailability and absorption [64]. A 54 % decrease in absorption of the antidepressant, sertraline, as measured by area-under-the-curve (AUC) testing, has been found as compared to pre-gastric bypass levels [65]. In another study, significantly decreased levels were measured in comparison to non-bypass controls (124.4 vs. 314.8 ng-hr/mL respectively) [66]. The decreased systemic exposure of the anti-depressant also had a clear clinical manifestation on the mood disorder of the patients. The absorption of the oral chemotherapy agent, imatanib has also been shown to decrease by a range of 46 % to 60 % after sleeve gastrectomy, though no clinical effect on disease progression was observed [49]. The evolution of pharmacokinetics after bariatric surgery, both gastric bypass and bilio-pancreatic diversion, was studied comparing the short-term (2 months) and long-term (2 years) absorption of the anti-cholesterol medication, atorvastatin. In the short term, there was an increase in absorption (AUC_8h_) by 1.6 ± 0.9, while the mean long-term/baseline ratio was lower at 0.8 ± 0.5 [67]. Bariatric surgery, both restrictive and malabsorptive, has generally demonstrated decreased oral drug absorption. Formal studies, short and long-term, will be essential to prevent possible under-dosing or inadequate immunosuppression.

### Pharmacokinetics of immunosuppressive medication after bariatric surgery

The standard three drug regimen of immunosuppression in kidney transplantation includes tacrolimus, mycophenolic mofetil and prednisone. The absorption of tacrolimus occurs primarily in the duodenum with secondary absorption in the small intestine and colon. Clinical dosing is guided by serum trough levels. The absorption of mycophenolate occurs in the stomach and duodenum. The measurement of the active plasma metabolite, mycophenolic acid (MPA) by AUC_24h_ is considered the most accurate way to measure systemic exposure, though practically dosing is determined by the clinical tolerance of side effects. An AUC in the range of 30 to 60 μg.h/mL has been described as the target to maximize immunosuppression while minimizing MPA-related side effects [68]. More recently, an extended release formulation of tacrolimus and an enteric-coated version of MPA have been marketed for increased compliance and fewer side effects.

Only one study has included pharmacokinetic testing in transplant patients who have had gastric bypass surgery. A higher dose was found to be necessary for sirolimus, tacrolimus and mycophenolate mofetil in a small cohort of six patients (four dialysis-dependent and two transplanted) as compared to a normal transplant population [69]. All other reports regarding immunosuppression after bariatric surgery have based their conclusions indirectly on changes in clinical dosing. A series of ten patients undergoing sleeve gastrectomy after transplantation, reported that two patients required an increased dose of tacrolimus, while another a decrease, based on serum trough levels [52]. Other case reports found a lower dose of the calcineurin inhibitor, cyclosporine was required [51], or no change at all [50]. Conclusions drawn from retrospective data of clinical dosing is subject to a multitude of other factors, including the evolution of the graft with respect to rejection and function, adverse effects of the medication, graft quality, co-morbidities and physician’s biases. Future pharmacokinetic studies will be necessary to gather proper data regarding the effect of bariatric surgery on immunosuppressive medication.

### Alternative clinical options for the management of obesity

There are limited clinical alternatives for the management of obesity in the transplant patient other than bariatric surgery and “diet and exercise”. One option is a minimally-invasive, robotic-assisted approach for the transplant operation in the obese patient to reduce the risk of surgical site infection [48]. However, this minimally-invasive approach only mitigates short-term complications and does not address the long term impact associated with obesity, including diabetes, metabolic syndrome and cardiovascular disease. Another approach is a simultaneous kidney transplantation and sleeve gastrectomy [70]. Combining these two operations has no obvious benefit other than limiting the patient to a single anaesthesia, while compounding the risks of both operation and the subsequent recovery afterwards. Otherwise, the last possibility would be to transplant regardless of obesity with the argument that a transplant provides a better outcome than remaining on dialysis [22], however this would ignore the negative long-term impact of obesity and disregard the possibility of risk-modification by bariatric surgery.

### Remaining questions regarding the treatment of obesity in kidney transplantation

Normally, it would be reasonable to expect that co-morbid diseases, such as diabetes or coronary atherosclerosis, be treated optimally prior to acceptance to the wait list, especially those felt to represent a modifiable risk. Some would even use this as a measure of compliance. Yet some diseases, such as obesity and tobacco addiction, often fall outside this expectation, possibly because they are more difficult to treat. As conservative weight loss appears to be largely ineffectual, a limit on BMI for candidacy imposed by a transplant program would make transplant practically inaccessible for obese patients. The only proven treatment is bariatric surgery. Would patients feel forced to undergo the surgery, albeit in the patient’s own best interest? If bariatric surgery were not offered as an option or if it was refused, would it be reasonable proceed with a kidney transplantation under sub-optimal conditions? Where does bariatric surgery fit in the transplant management algorithm in the patient-centered medicine environment? Could the operation be deferred to after transplantation, if the patient were willing to accept the peri-operative and post-transplant risks of obesity? Many of these questions are difficult to answer.

### Future directions

Prospective clinical studies of bariatric surgery in the transplantation will be necessary to answer several key questions. Initially, the safety and efficacy of bariatric surgery will have to be demonstrated, including the sustainability of weight control over the long term (>5 years) and the effect of bariatric surgery on post-transplant weight gain. Additionally, the role or indication of bariatric surgery in the obese transplant population will be defined by a relative risk reduction in operative complications, post-transplant diabetes, cardiovascular disease, graft loss and patient mortality. Any longitudinal studies should include a wide breadth of anthropometric data which will help in determining the strongest prognostic measure of the impact of obesity and weight loss by bariatric surgery. Other potential research could look at the impact of surgical weight loss on the obesity-related mechanisms of chronic renal disease and systemic inflammation.

## Conclusions

The risks of surgical complications, graft failure and patient death that are associated with obesity in the kidney transplant population cannot be understated. As a result, most transplant programs use BMI in the selection criteria for wait-listing. For such patients, obesity simply cannot go untreated, to give them hope of an eventual transplantation and for their health in general.

Unfortunately, conservative weight management through diet and exercise is inefficient for the vast majority of candidates and recipients, as it has been in the general population. Several retrospective series of bariatric surgery in the transplant population have shown dramatic and rapid weight loss with promising secondary benefits to diabetes and hypertension.

Prospective clinical studies will be necessary to properly evaluate the peri-operative safety, the effect on pharmacokinetics of immunosuppression and the long-term durability of bariatric surgery in the transplant population. Secondary benefits to diabetes, hypertension and even CRF will be important to consider in the design of such studies. Bariatric surgery has the hope of being the efficient weight management strategy that transplant specialists have longed for.

## Abbreviations

BMI: Body mass index
CRF: Chronic kidney disease
ESRD: End stage renal disease
BMI: Body mass index
LGB: Laparoscopic gastric bypass
LDS: Laparoscopic duodenal switch
LSG: Laparoscopic sleeve gastrectomy
AUC: Area under the curve
RR: Relative risk
CI: Confidence interval
USRDS: United States renal data system
